# Green synthesis of *Colocasia esculenta*-based silver nanoparticles: characterization and transdermal delivery for anti-inflammatory applications

**DOI:** 10.3389/fphar.2025.1611507

**Published:** 2025-08-14

**Authors:** Xiaobo Wang, Wanjuan Wang, Areeba Ejaz, Maira Mehmood, Nadeem Ahmad, Imran Nazir, Yasser Shahzad, Tianshuo Bai

**Affiliations:** ^1^ Department of Dermatology, Hejin Wang Xiaobo Dermatology Clinic, Hejin, China; ^2^ Department of Dermatology, The Second Affiliated Hospital of Xi’an Jiaotong University (Xibei Hospital), Xi’an, China; ^3^ Department of Pharmacy, COMSATS University Islamabad, Lahore campus, Lahore, Pakistan; ^4^ Department of Emergency, XD Group Hospital, Xi’an, China

**Keywords:** *C. esculenta* extract, green synthesis, anti-inflammatory, transdermal patches, *ex vivo* permeation

## Abstract

The current study aims to develop chitosan transdermal patches containing AgNPs synthesized and characterized from *C. esculenta* to reduce inflammation. Silver nanoparticles (AgNPs) were prepared utilizing the *C. esculenta* extract and characterized through Fourier transform infrared spectroscopy (FTIR), scanning electron microscopy (SEM), X-ray diffraction (XRD), zeta potential measurements, and particle size analysis. The AgNPs were incorporated into chitosan-based transdermal patches, which were assessed for physicochemical properties, dermal compatibility, and anti-inflammatory effectiveness. *In vitro* protein denaturation and *in vivo* carrageenan-induced paw edema models evaluated anti-inflammatory efficacy. Characterization verified that the AgNPs were stable and spherical. The transdermal patches were biocompatible, had uniform thickness, and were extremely adhesive. The anti-inflammatory effects of AgNP-loaded patches were found to be superior to those of diclofenac sodium in *in vivo* studies, while *in vitro* studies revealed a notable inhibition of protein denaturation (p < 0.05). The current study offers new evidence that transdermal drug delivery systems derived from *C. esculenta* can effectively and sustainably manage inflammation.

## 1 Introduction

Nanotechnology is employed in the fabrication of various functioning systems at the molecular level. These systems exhibit specific electrical, physical, and optical features, making them valuable in a wide array of domains such as biology and material science (Bamrungsap et al., 2012). Nanotechnology involves the synthesis of nanoparticles (NPs) in the size range of 10–1,000 nm, in which active pharmaceutical ingredient (API) is entrapped or encapsulated, thus experiencing many advancements in science due to its invaluable applications in medicine, catalysis, material science, and drug delivery (Mohanraj and Chen, 2007). Previously, various metal NPs and their complexes in the form of oxides, sulfides, fluorides, hydroxides, chlorides, and phosphates have been formulated and employed in various applications (Paramasivam et al., 2017; Ramos et al., 2017; Rasouli et al., 2018). Various physical, chemical, and biological methods can be employed in the synthesis of nanomaterials. As these conventional methods include the use of toxic materials, green alternative approaches have been explored to minimize the use of toxic materials (Adeyemi et al., 2022; Câmara et al., 2022). Some of these approaches require NP synthesis by employing the plant extract, which is simple, ecofriendly, sustainable, non-hazardous, and cost effective (Bharadwaj et al., 2021; Gupta et al., 2023).

Nanoparticles can be synthesized from a variety of sources, including noble metals like silver, platinum, palladium, gold, titanium dioxide, and zinc oxide. Ionic silver has taken the place of silver (Ag) to formulate the bulk coins and is currently employed for the formulation of colloidal silver NPs (Chandoliya et al., 2024; El-Seedi et al., 2024). It is also utilized as the silver nitrate to present the antimicrobial properties. Silver NPs exhibit enhanced antimicrobial effects due to an increased surface area, which can interact with the microbial species efficiently. Silver is the widely used element of choice for NP synthesis in the fields of medicine, living organisms, and biological systems. Thus, the combination of reducing agents with silver to form NPs with better activity and efficacy is an area of interest (K. Singh et al., 2014). Physical and chemical methods are commonly employed for the synthesis of NP with more stability as colloidal dispersion in organic solvents or water. However, chemical methods employed for the NP synthesis use noxious chemicals that affect plants, animals, and the overall environment. Thus, there is a need to explore new approaches to synthesize the NPs in a pollution-free environment to ensure a pollution-free and healthy environment.

Nanobiotechnologists are striving to explore new green approaches to replace the existing hazardous NP synthesis techniques. Green biology involves attractive domains such as green technology, green engineering, green polymerization, silver chemistry, wastewater treatment, bio-composites, and particle technology (Kharissova et al., 2009). Green chemistry for silver NP synthesis is ecofriendly, simple, less time consuming, cost effective, and favors the large-scale production of NPs by utilizing a common capping, reducing, and stabilizing agent (Lade and Patil, 2017). Green approaches to NP formulation include natural products, table sugar, polyphenols, glucose, amino acids, and plant extracts. Plant leaf extracts are widely utilized for the reduction of AgNO_3_ to Ag, as they contain various functional groups such as carboxylic acid, aliphatic amine, amines, alkanes, alkynes, alcohol, nitro-compound, saturated aldehyde, phenols, and glutathione (Balavandy et al., 2014; B. Kumar et al., 2015). Protective foods include the green leafy vegetables harboring a spectrum of bioactive compounds and micronutrients that boost the immune system and prevent diseases. The distinctive therapeutic index of bioactive compounds present in the leafy vegetables has been emphasized in various studies (Anand et al., 2019; Mehmood and Zeb, 2020).

*Colocasia esculenta* (*C*. *esculenta*) Shott, generally recognized as “*taro,*” is an annual herbaceous plant of the *Araceae* family (Goncalves et al., 2013). It is referred to as tabbia, cocoyam, dasheen, or eddoe in different parts of the world (Dilek and Bilgiçli, 2021). *C*. *esculenta* is native to Southeast Asia and cultivated in the tropical and subtropical regions (Chand et al., 2021). Depending upon its genotype, its leaves are heart shaped with or without blotches, lines, and spots. Its color varies from light green to dark purple. *C*. *esculenta* exhibits various medicinal properties such as antidiabetic, antioxidative (Sudhakar et al., 2020), anticancer (Pereira et al., 2021), anti-inflammatory (Baro et al., 2023), anti-hypersensitive (Esposito et al., 2024), and antimicrobial (Sudhakar et al., 2020). Its leaves contain various sugars (glucose, fructose, and sucrose, etc.), vitamins (thiamin, niacin, and riboflavin), and minerals (iron, potassium, calcium, and copper) to cause rapid oxidation, explaining their enhanced potential as reducing agents (K. K. P. Kumar et al., 2019).

In the current study, we formulated the NPs from *C*. *esculenta* by reacting them with AgNO_3_ and adopting green synthesis. Formulated NPs were characterized for shape, particle size, zeta potential, X-ray diffraction (XRD), and FTIR analysis. Optimized NP formulations were then integrated into transdermal patches synthesized from chitosan. NP-harboring patches were analyzed for physical character, adhesion, skin irritation, moisture content, thermal analysis, and sensitization. These patches were also subjected to *in vivo* anti-inflammatory studies and toxicity studies.

## 2 Materials and methods

### 2.1 Materials

*C*. *esculenta plant* leaves were procured from the local market of Lahore, Pakistan. The plant material was authenticated and verified under voucher No. GC.Herb.Bot.4184 by comparison of the morphological features with authenticated specimens in the herbarium by the taxonomist at the Department of Botany, Government College University, Lahore, Pakistan. Silver nitrate (AgNO_3_), acetic acid, glutaraldehyde, glycerol, sodium hydroxide (NaOH), ortho-phosphoric acid, potassium dihydrogen phosphate (KH_2_PO_4_), and methanol (HPLC grade) were procured from Sigma-Aldrich Co., St Louis, MO, USA. Low-molecular weight chitosan (CAS No: 9012-76-4), with a molecular weight of 50–190 kDa, ∼75%–85% degree of acetylation, and purity >98%, was also procured from Sigma-Aldrich (USA). All other reagents and materials used in this study were of analytical grade.

### 2.2 Methods

#### 2.2.1 Formulation of *C*. *esculenta* extract

*C. esculenta* leaves were washed with Milli-Q water to detach dust and attached impurities. Leaves were subjected to air drying under shade at room temperature (25 °C) for 3–4 days. Afterward, 40 g of dried leaves was sliced into small pieces and boiled in 500 mL of distilled water while stirring continuously via a magnetic stirrer at 60 °C for 30 min. The resulting mixture was cooled to room temperature (25 °C), filtered through Whatman No. 1 filter paper, and subjected to vacuum filtration assembly to acquire the clear extract. The pH of the prepared extract was recorded (∼6.8–7.0), and it was stored in amber-colored bottles at 4 °C for further use (K. K. P. Kumar et al., 2019).

#### 2.2.2 Preparation of *C. esculenta* extract-based AgNPs

Silver NPs were prepared according to the method described by Lade and Patil (2017) with small modifications. The aqueous AgNO_3_ solution (8 mM) and varying concentrations of aqueous extract of *C. esculenta* were mixed in Erlenmeyer flasks to synthesize the silver nanoparticles, as shown in Table 1. The pH of the reaction mixture was adjusted to ∼8.0 with 0.1 M NaOH, and reactions were carried out at 70 °C in the water bath for different time intervals (30 min, 45 min, and 60 min) with continuous stirring. The color of the mixture changed from pale yellow to brown, indicating the Ag^+^ reduction to Ag° nanoparticles. The colloidal solution was centrifuged at 10,000 rpm for 10 min. The NP pellet was harvested and rinsed twice with distilled water, followed by freeze-drying in a benchtop freeze dryer (LYO60B-1 PT Bioevopeak, China). The NP pellet was then stored at 4 °C for further use.

**TABLE 1:** Composition of different formulations of *C. esculenta* NPs.

Composition	F1	F2	F3	F4	F5	F6
*C. esculenta* extract (mL)	5	10	15	20	25	30
8 mM AgNO_3_ volume (mL)	95	90	85	80	75	70

#### 2.2.3 Preparation of silver NP-containing chitosan patches

Varying concentrations (1%–3%, w/v) of chitosan (CS) were dissolved in 1%–2% acetic acid solution (v/v) individually to make the CS solutions to achieve the desired thickness and mechanical properties of the patch. The solution was stirred under magnetic stirring for several hours (12–24 h) to achieve the complete dissolution of the CS in the solution. Polyethylene glycol (PEG-400, 1%–2% v/v) was added to the CS solution as a plasticizer to enhance the mechanical properties and flexibility of the formulated patch. Similarly, 100–300 mg of PVP-k30 was added to enhance the mechanical strength, skin adhesion, and stability of the prepared patch. Subsequently, 5% (w/v) of *C. esculenta*-derived AgNP product (lyophilized powder redispersed in distilled water) was mixed into the CS solution. The resulting mixture was poured into the casting molds or Petri dishes to generate the thin layer. The solution was then dried in the drying oven under controlled conditions at 30 °C–40 °C for 24 h. After drying, the formed patches/films from the molds were peeled off carefully (Sabbagh and Kim, 2022). Various formulations of patches manufactured with *C. esculenta* AgNPs are presented in Table 2. Regarding quantification in the final patch, it contained AgNPs (5% w/v) as per the formulation shown in Table 2. The AgNP concentration was kept uniform based on the controlled batch conditions and the synthesis protocol, ensuring a consistent AgNP concentration across the patches.

**TABLE 2:** Composition of different formulations of chitosan-based transdermal patches with varying concentrations of *C. esculenta* extract-based NPs (Mean ± standard deviation (SD)).

Composition	F1	F2	F3	F4	F5	F6	F7
Chitosan (mL)	20	25	30	25	25	25	25
PEG 400 (mL)	1	1.5	2	2	2	2	2
PVP-k30 (mg)	100	200	300	200	200	200	200
NPs of *C. esculenta* (%w/v) from Table 1	5	5	5	5	5	5	Nil
Diclofenac Na (mg)	Nil	Nil	Nil	Nil	Nil	Nil	50

#### 2.2.4 Characterization of *C. esculenta* AgNPs and CS transdermal patches

##### 2.2.4.1 FTIR spectral analysis

Fourier transform infrared spectroscopy (FTIR, JASCO FT/IR 6300 spectrometer) analysis was performed to analyze the chemical structure and functional groups of the *C. esculenta* plant extract and interactions of different functional groups during the formation of *C. esculenta*-AgNPs *and C. esculenta*-AgNP-loaded transdermal patches. Samples were prepared in KBr pressed pellets, and spectra were recorded in the 4,000–400 cm^−1^ range with a spectral resolution of 4 cm^−1^ from the average of 100 spectra in the absorbance mode.

##### 2.2.4.2 UV spectrophotometry

UV–visible spectroscopy was conducted with a VWR UV-1600PC UV/Vis spectrophotometer. Samples were evaluated in the 400–900 nm spectral range.

##### 2.2.4.3 Scanning electron microscopy (SEM)

SEM studies of *C. esculenta* extract AgNPs and prepared transdermal patches were conducted to analyze the surface morphology. Samples were prepared as a fine powder and coated with gold to be analyzed using SEM (Quanta FEG250) at a high voltage (20 kV) of electron beam to analyze the surface morphology.

##### 2.2.4.4 Powder X-ray diffraction (XRD) studies

XRD studies of the dried extract of *C. esculenta* and prepared AgNPs of the *C. esculenta* extract were performed by using XRD (X-Pert, PAN analytical, Netherlands), and diffractograms were detailed at 2*θ* = 5–70^°^ to analyze the crystalline or amorphous nature of the samples.

##### 2.2.4.5 Zeta potential and particle size analysis

Light scattering studies were performed with a He–Ne laser operating at a wavelength of 633 nm at 25 °C by using a ZETASIZER (Nano ZS; ZEN3600, Malvern). The intensity size distributions were acquired from the evaluation of the correlation functions by the Multiple Narrow Modes algorithm. The prepared samples (AgNPs, AgNPs 200, 300, and 400) were diluted 1:20 with distilled water to remove the primary charge, followed by ultrasound for 10 min to prevent agglomeration. A 2-mL aliquot of the sample was transferred in disposable cuvettes of 10 mm diameter. The experiment was repeated three times to analyze the repeatability of the results. Both surface charge and stability of the particles were determined by measuring the zeta potentials (SZ-100, Horiba Scientific, Kyoto, Japan). Formulated NPs were diluted with deionized water (1/10, w/v) and transferred into the measurement cell for analysis (n = 5).

#### 2.2.5 Characterization of C. *esculenta* AgNP-loaded chitosan patches

All prepared formulations of C. esculenta AgNPs loaded CS patches were characterized for the below mentioned parameters:

##### 2.2.5.1 Physical appearance

The physical appearance of formulated patches was analyzed to check the irregularities in the form of wrinkles, air bubbles, or roughness. Color uniformity across the entire patch was also ensured, as discoloration of the patch may lead to degradation. Similarly, patches were also observed for the cloudiness, as it is a sign of material change. The batch number and expiry date were also monitored.

##### 2.2.5.2 Thickness uniformity

Prepared patches were subjected to thickness measurement by employing an electronic caliper with a thickness count of 0.01 mm. Thickness uniformity was ensured by measuring the thickness at three different positions of the film, and the average thickness of the measured readings was taken.

##### 2.2.5.3 Adhesive properties

Adhesiveness of the patch was evaluated, and even distribution of the adhesive layer over the patch was ensured to eradicate the chance of defect or lump formation on the patch. Peel adhesion and tackiness were also evaluated by the gentle pressing of the patch on the clean surface.

##### 2.2.5.4 Moisture content

Prepared patches were subjected to a moisture test to evaluate the moisture content present in the patches, as excess moisture can affect the drug stability and appearance of the patch.

##### 2.2.5.5 Skin irritation and sensitization tests

Skin irritation caused by patch application can be evaluated by the Draize patch test. Skin irritation causes visible changes in the skin, such as erythema, edema, or redness, after multiple applications of the patch over the skin.

##### 2.2.5.6 Uniformity of weight

The weight of the prepared patch was measured after cutting the patch into multiple pieces of 1 × 1 cm^2^ size each, and the average weight of the patch was calculated.

##### 2.2.5.7 Folding endurance

The folding endurance of the patches was measured manually. Briefly, a patch strip of 2 × 2 cm^2^ size was folded at the same point repeatedly until broken, and the folding endurance was calculated by counting the number of folds a patch film could withstand until it was broken.

##### 2.2.5.8 Tensile strength

Prepared patches were also subjected to tensile strength measurement using a Universal Testing Machine (UTM 100-500KN, Testometric Inc., UK). The apparatus consisted of 5N load cells and operated at room temperature. A 4 × 1 cm^2^ sample of the test film was prepared and placed between the lower and upper cell grips, followed by force application. The force was increased gradually until the film was broken. Tensile strength was measured in Kg and expressed as follows:

Tensile strength=tensile load at break/cross sectional area.

#### 2.2.6 *Ex vivo* permeation study

*Ex vivo* investigations of prepared patches and nanoparticles were conducted on the skin of rats. The procedure to extract the rat skin received approval from the Institutional Animal Ethical Committee (Ref. no: PHM.Eth/Lhr-05/08-24). Male Sprague–Dawley rats weighing 200–250 g were sacrificed via cervical dislocation. Hairs of rats were excised from the dorsal area utilizing an electric trimmer, followed by skin excision surgically. Skin was rinsed with a 0.9% sodium chloride solution and stored at −20 °C ± 1 °C. The rat’s skin was subsequently positioned between the donor and receptor compartments of the Franz diffusion cell. The phosphate buffer (pH 7.4) was filled in the recipient compartment, and the temperature was set at 37 °C ± 2 °C. The receiving medium was agitated at 100 revolutions per minute. The donor partition was filled with a nanoparticle patch exhibiting 5 mg of the drug. Subsequently, 2-mL aliquots were collected at designated time intervals (0.5 h, 1 h, 1.5 h, 4 h, 8 h, 12 h, 16 h, and 24 h), and the medium was replenished with fresh buffer to preserve the sink conditions. The samples were subjected to UV–Vis spectrophotometry at a wavelength of 425 nm for further analysis (Nawaz et al., 2022).

#### 2.2.7 *In vitro* anti-inflammatory study (egg albumin denaturation study)

The *in vitro* anti-inflammatory effect was assessed through a protein denaturation test utilizing egg albumin. We performed the egg albumin denaturation assay following the protocol described in previously conducted studies (Abbas et al., 2021; Mizushima and Kobayashi, 1968) with minor modifications. This method involved mixing a test sample (2 mL) with a concentration of 100–400 g/mL, egg albumin (0.2 mL), and phosphate buffer saline, pH = 6.5 (2.8 mL). The reaction mixture was incubated at 37 °C for 20 min, followed by heating at 70 °C for 5 min. When the temperature dropped to room temperature, the absorbance was assessed at 660 nm using a spectrophotometer. Phosphate buffer served as the control, while diclofenac Na was employed as the standard drug. Percentage inhibition was calculated using the following equation:

$$\%\mathrm{Inhibition of denaturation}=100\;*\;\left(\text{AC}-\text{AS}\right)/\text{AC}$$

where AC is the absorption of the control, and AS is the absorption of the test sample.

#### 2.2.8 *In vivo* anti-inflammatory studies

##### 2.2.8.1 Animals

The subjects employed for *in vivo* anti-inflammatory studies were female Wistar rats (140–190 g). Animals were acquired from the laboratory Animal Services Division of the Central Drug Research Institute. Subjects were reserved in a polyacrylic cage (22.5 × 37.5 cm) under the standard housing environment with humidity 60%–65% and room temperature 24°C–27°C. Water and food were provided *ad libitum,* but food was restricted 1 h prior to the behavioral studies. The anti-inflammatory study protocol was approved by the Institutional Animal Ethical Committee (Ref. no: PHM.Eth/Lhr-05/08-24), and studies were conducted according to the Committee for the Purpose of Control and Supervision of Experiments on Animals (CPCSEA) guidelines regarding the care of experimental subjects.

##### 2.2.8.2 Paw edema induced by carrageenan

Experimental subjects were categorized into four groups (n = 5). Group I of the experimental subjects was considered as a normal control without induced edema by carrageenan. 0.1 mL of 1% carrageenan in normal saline was administered via the sub-plantar route in the left-hand paw of the subjects of groups II, III and IV. Measurements of the paw volume were taken with the plethysomometer at 0 h, 0.5 h, 1 h, 2 h, and 3 h after administering the carrageenan. Group II of the experimental subjects received the normal saline 3 mL/kg body weight, intraperitoneal, and is referred to as the saline control, while Group III received the *C. esculenta* plant extract NPs 5% w/v in the transdermal patch. Group IV received the transdermal patch loaded with diclofenac Na (8 mg/film) for the treatment of paw edema caused by carrageenan administration. The formula employed to calculate the % paw edema inflammation is as follows:

% Inhibition=control-treated \/control*100.

Furthermore, blood samples from the experimental subjects were also subjected to the hematological analysis to assess the hematological parameters, including, hemoglobin concentration, platelet count, red blood cell count, and white blood cells count, via an automated hematology analyzer (Sysmex KX-21). Blood samples were analyzed within 2 hours of collection from the subjects.

#### 2.2.9 Statistical analysis

All statistical analyses were performed by GraphPad Prism v. 9.0 (GraphPad Software, USA), and data were expressed as mean ± standard deviation (SD). One-way ANOVA followed by Tukey’s multiple comparison test was selected to compare multiple formulations in the evaluation of physicochemical parameters of transdermal patches. In addition, a paired t-test was employed to compare results before and after treatment for Group II, while one-way ANOVA was used for intergroup comparison in the evaluation of biochemical parameters. An unpaired t-test was applied to compare the control with the treatment groups. Results were considered statistically significant at p < 0.05.

## 3 Results and discussion

### 3.1 Synthesis of C. *esculenta* AgNPs

**FIGURE 1:**
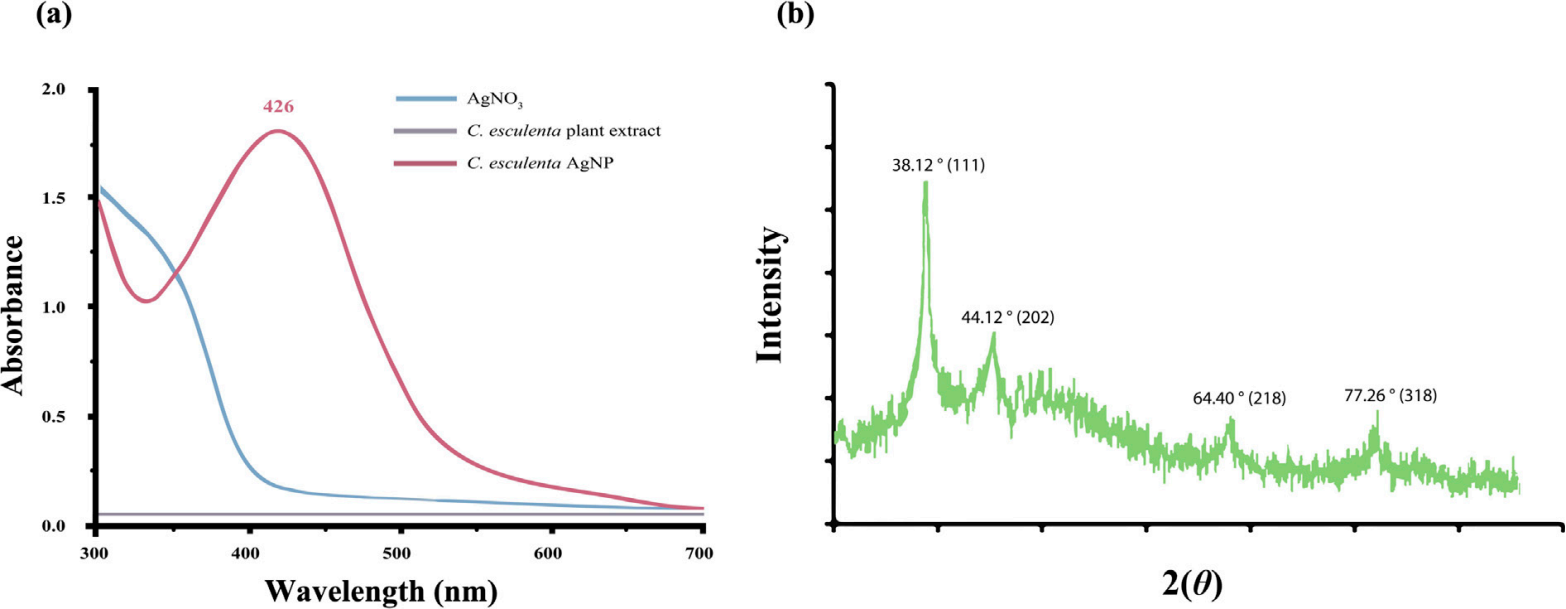
**(a)** UV analysis of synthesized *C. esculenta* AgNPs and **(b)** XRD analysis of *C. esculenta* AgNPs.

*C. esculenta* AgNP synthesis is generally divided into three phases: nucleation, nuclei conversion into seeds, and, eventually, seed growth into nanocrystals (Abbas et al., 2021). To formulate *C. esculenta* AgNPs, *C. esculenta* extract was added to synthesize AgNPs. Ag^+^ ions in AgNO_3_ were reduced to Ag atoms by the reducing ingredients in the plant extract, depicted by the change of color from green to bright reddish-brown upon stirring. In addition, *C. esculenta* AgNPs are favorable for the calorimetric analysis due to their bright color and high molar absorbance (Figure 1a).

### 3.2 UV spectrophotometry

UV spectrophotometry was employed to analyze the optical characteristics depending upon the size effects to confirm the synthesis of metal NPs. The color of *C. esculenta* AgNPs was evaluated using UV–Vis. Various color changes were observed during the synthesis of *C. esculenta* AgNPs, and specific absorption was shown by *C. esculenta* extract and synthesized *C. esculenta* AgNPs (Figure 1a). Ag ions were reduced by the extract of *C. esculenta* and assessed by UV–Vis spectroscopy. No peak for silver salt and plant extract was observed in the range of 380–460 nm, but an obvious peak appeared for the synthesized AgNPs in the range of 380–460 nm without any noise, indicating the surface plasmon resonance characteristic of the aggregated and spherical AgNPs. Results revealed the surface plasmon resonance (SPR) bands in the visible region (∼426 nm) showing the synthesis of AgNPs as shown in Figure 1a. As AgNPs derived from different plant extracts exhibit different absorption peaks, their SPR bands are usually found in the absorption range of 350–450 nm (Beg et al., 2018; Francis et al., 2019). 

### 3.3 Effect of silver nitrate concentration

**FIGURE 2:**
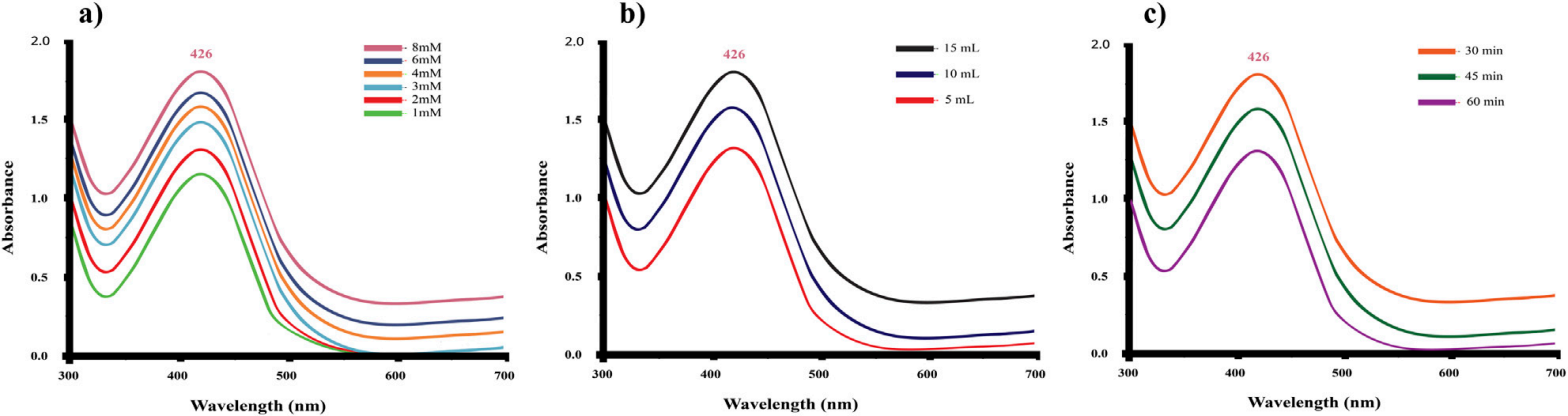
**(a)** Effect of AgNO_3_ concentration on the synthesis of *C. esculenta* AgNPs; **(b)** effect of *C. esculenta* extract concentration on the synthesis of *C. esculenta* AgNPs; **(c)** effect of reaction time on the synthesis of *C. esculenta* AgNPs.

AgNO_3_ concentration affects the synthesis of AgNPs (Figure 2a) and the SPR peak position. According to the UV spectrum, the highest peak appeared at the AgNO_3_ concentration of 8 mM, expressing the optimal formulation concentration of AgNO_3_ to synthesize the AgNPs. Due to the increase in Ag salt concentration, a portion of Ag^+^ ions is reduced to Ag atoms with the plant extracts, which in turn serves as a starting point of nucleation. Consequently, the remaining Ag^+^ is reduced, leading to the generation of more AgNPs and increased absorbance. NP size was increased due to the formation of clusters by the growth of individual nuclei, causing a red shift in the absorption peaks of the UV spectrum toward higher wavelengths. Results revealed that the SPR peak position remained unchanged by an increase in Ag+ within the AgNO_3_ concentration range of 2–8 mM. However, previous studies described the competition and aggregation effect of NPs contributed toward the shifting of the SPR band, which was not affected by increased concentration of Ag^+^ (Zaheer, 2018). In addition, AgNO_3_ concentration (10 mM) caused aggregation of the NPs due to the increased collision frequency of the Ag^+^ ions. Exposure to a high concentration of AgNO_3_ led to the deposition of silver salts on AgNPs, leading to the appearance of an unclear surface (Park et al., 2012). These results showed that 8 mM of AgNO_3_ is the optimum concentration to synthesize the *C. esculenta* AgNPs, avoiding any aggregation between the particles.

### 3.4 Effect of the *C. esculenta* extract concentration

The concentration of *C. esculenta* extract affects the absorbance (Figure 2b). An increased extract concentration increases the absorbance, leading to increased production of AgNPs due to secondary reduction of Ag^+^ ions (Satpathy et al., 2018). The nucleation process is facilitated by the higher concentration of reducing agents. One study demonstrated that decreased concentration of extract resulted in the synthesis of a low number of AgNPs, and some of them exhibited anisotropic and irregular nanostructures (Sathishkumar et al., 2010). To achieve the optimum yield of *C. esculenta* AgNPs, 15 mL of *C. esculenta* extract was found to be appropriate in size (˂100 nm) and morphology.

### 3.5 Effect of the reaction time

*C. esculenta* AgNP synthesis was accomplished within 30 min of the reaction at 70 °C. A slow rate of NP nucleation with unchanged absorption peaks was observed with a rise in reaction time (Figure 2c). In addition, the width and position of SPR peaks also remained constant, showing that increased reaction time does not alter the morphology and size of the AgNPs. However, decreased absorption intensity was observed with an extension of reaction time up to 60 min. This decrease in absorption intensity is attributed to instability and aggregation of nanosilver (S. P. Singh et al., 2021). Mittal et al. revealed that maximum absorption occurred after 12 h of reaction time, followed by aggregation of particles after 24 h, by employing *Syzygium cumini* fruit extracts in the synthesis of AgNPs (Mittal et al., 2014). Based on the use of *C. esculenta* for the synthesis of AgNPs, the time expenditure can be minimized to within 45 min.

### 3.6 FTIR spectral analysis of *C. esculenta* AgNP

**FIGURE 3:**
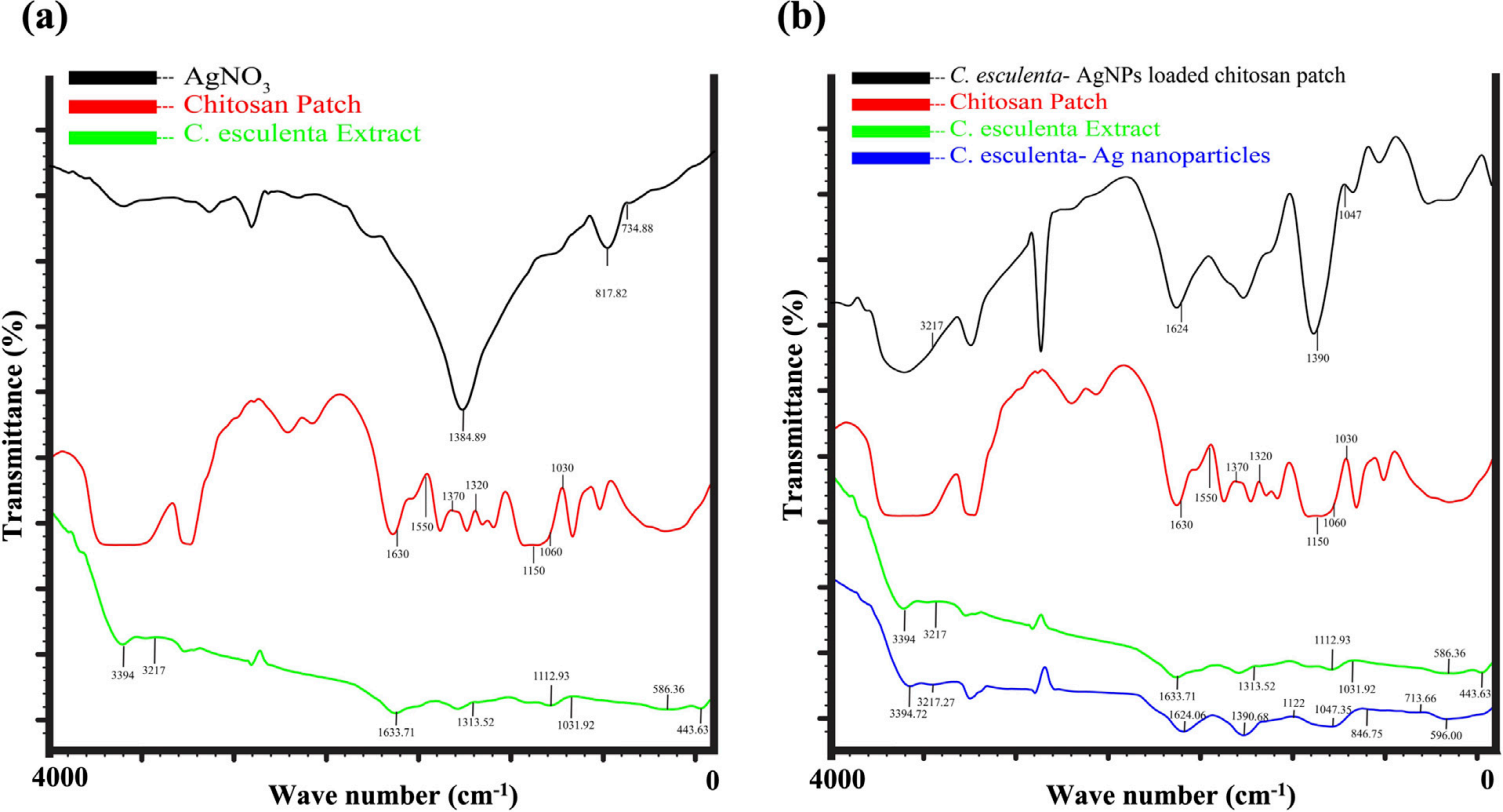
TIR analysis of **(a)** FTIR analysis of AgNO3, CS patch, and C. esculenta extract **(b)** C. esculenta Ag-NPs loaded CS patch, Formulated CS patch, C. esculenta extract and C. esculenta Ag-NPs.

FTIR analysis revealed the identification of phytochemicals of the extract involved in the coating of AgNPs by depicting the molecular vibrations (bending, twisting, and stretching of the chemical bonds) of the sample in particular infrared regions (Mondal et al., 2023). The FTIR spectra of AgNO_3_ yield various peaks at 734 cm^−1^, 817 cm^−1^, and 1,384 cm^−1^ due to the bending and asymmetric stretching vibrations of nitrate ions. *C. esculenta* plant extract showed significant absorption at the range from 443–586 cm^−1^, 1,031 cm^−1^, 1,112 cm^−1^, 1,313–1,633 cm^−1^, and 2,401–3,832 cm^−1^ due to C-C or C-O bending, C-O or C-O-C stretching, C-H bending, and C = O stretching and O–H and N–H stretching, respectively (Figure 3a). These absorption peaks identified the presence of polysaccharide, phenolic, and flavonoid compounds within *C. esculenta* extract. However, the spectra of AgNP formulated by employing *C. esculenta* plant extract showed various significant and minor shifts within the absorption peaks. The reductions in the nitrate peaks at 734 cm^−1^, 817 cm^−1^, and 1,384 cm^−1^ confirm the proper consumption of AgNO_3_ (Figure 3a). Furthermore, the shifting of plant extract peaks from 1,031 cm^−1^, 1,122 cm^−1^, and 1,633 cm^−1^ to 1,047 cm^−1^, 1,390 cm^−1^, and 1,624 cm^−1^ indicates interaction between phytochemicals and Ag ions, as shown in Figure 3b. The appearance of new characteristic peaks at 596 cm^−1^, 713 cm^−1^, 846 cm^−1^, 1,047 cm^−1^, 1,390 cm^−1^, and 1,624 cm^−1^ confirmed the binding of phytochemicals with nanoparticles. Broadening of the O-H and N-H bands at 3,217 cm^−1^ and 3,394 cm^−1^ provides evidence of the stabilization of phytochemicals due to the generation of hydrogen bonding and hence provides proper capping of these chemicals.

### 3.7 Scanning electron microscopy (SEM)

**FIGURE 4:**
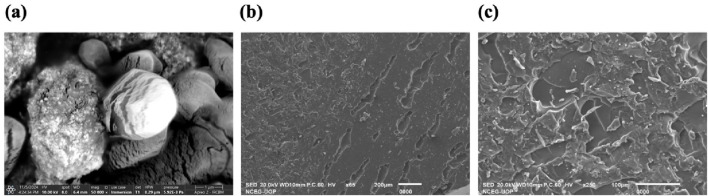
SEM analysis of **(a)** *C. esculenta* AgNPs; **(b)** formulated CS patch; **(c)** *C. esculenta* AgNP-loaded CS patch.

SEM was employed to explore the morphology of AgNPs, as morphology plays an important role in the characteristics and potential applications of NPs in biomedicine and nanotechnology. Spherical aggregated clusters of AgNPs were observed (Figure 4a) in the SEM images, and these results are in concordance with the previous studies (Arya et al., 2024; Okaiyeto et al., 2021).

### 3.8 Powder X-ray diffraction (XRD) studies

XRD analysis of the prepared *C. esculenta* AgNPs demonstrated distinct diffraction peaks at 2θ values of 38.12°, 44.12°, 64.40°, and 77.26°, corresponding to the 111, 202, 218, and 318 lattice planes, respectively (Figure 1a). These results are the property of the face-centered cubic (FCC) structure of the metallic silver and are consistent with JCPDS file No. 04-0783. These intense and sharp peaks verify the crystallinity of the nanoparticles and preferential growth direction in the 111 plane (Ryu et al., 2022; Yi et al., 2014).

### 3.9 Zeta potential and particle size analysis

**FIGURE 5:**
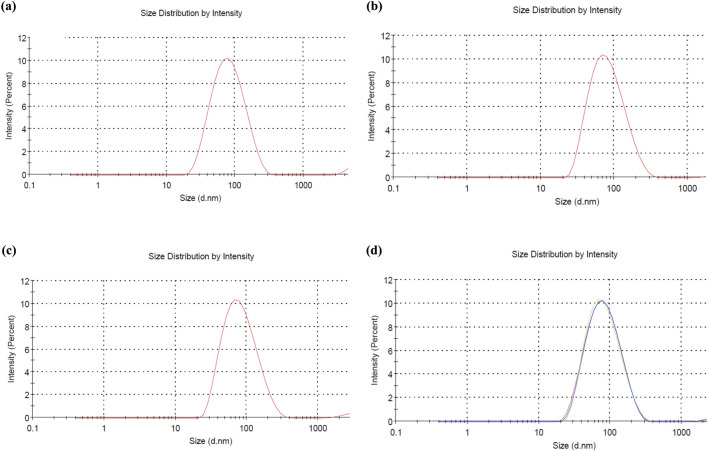
Size analysis of **(a)** F1 formulation of *C. esculenta* AgNPs, **(b)** F2 formulation of *C. esculenta* AgNPs, **(c)** F3 formulation of *C. esculenta* AgNPs, and **(d)** collective size of all formulations of *C. esculenta* AgNPs.

The zeta potential, particle size, and polydispersity index (PDI) of *C. esculenta* AgNPs were investigated. The size distribution of AgNPs was measured by dynamic light scattering (DLS), and the particle size of AgNPs was evaluated to be 90.17 ± 12.65 nm with a PDI value of 0.281 ± 0.07, which shows the polydispersity (Figure 5). The measured zeta potential value was −31.6 ± 1.26 mV, indicating the surface charge to deliver the strong repulsion between nanoparticles and minimize the agglomeration risk. This value suggests that the nanoparticles exhibit a stable electrical double layer and colloidal stability. In addition, the low PDI (0.281 ± 0.07) indicates the uniform size distribution, reduced aggregation and sedimentation, and enhanced colloidal stability. These findings confirmed that *C. esculenta-*mediated AgNPs exhibit dispersion stability in the aqueous media (José Pochapski et al., 2021; Riaz et al., 2021). Particles are considered stable with the zeta potential values below −30 mV or exceeding +30 mV (Chinni et al., 2021). In this regard, *C. esculenta* AgNPs may avoid particle aggregation and retain stability in solution owing to the electrostatic repulsion between the nanoparticles.

### 3.10 Characterization of C. esculenta AgNPs loaded CS patches

#### 3.10.1 FTIR spectral analysis of CS transdermal patches

FTIR analysis was performed to verify the successful integration of *C. esculenta* AgNP into CS transdermal patches. The spectral analysis of *C. esculenta* AgNP, the chitosan patch, and the *C. esculenta* AgNP-loaded chitosan patch demonstrated notable differences. Significant absorption peaks in *C. esculenta* AgNP were observed at 596 cm^−1^, 713 cm^−1^, 846 cm^−1^, 1,047 cm^−1^, 1,390 cm^−1^, 1,624 cm^−1^, 3,217 cm^−1^, and 3,394 cm^−1^, indicative of metal–ligand vibrations and biomolecule capping. The CS patch displayed peaks at 580 cm^−1^, 837 cm^−1^, 1,082 cm^−1^, 1,647 cm^−1^, and 3,331–3,468 cm^−1^, corresponding to the CS and PEG functional groups employed in the patch formation, as summarized in Figure 3A. Upon the incorporation of *C. esculenta* AgNP into the patch, novel peaks appeared at 569 cm^−1^, 650 cm^−1^, 840 cm^−1^, 945 cm^−1^, 1,099 cm^−1^, 1,377 cm^−1^, 1,639 cm^−1^, and 2,156 cm^−1^, signifying Ag-polymer interactions. Displacements in O–H or N–H stretching (3,427 cm^−1^) and the emergence peaks at 3,716 cm^−1^, 3,867 cm^−1^, and 3,992 cm^−1^ validated the augmentation of hydrogen bonding and molecular reconfiguration. The absence of peaks at 1,251 cm^−1^, 1,296 cm^−1^, and 1,357 cm^−1^ in the CS patch spectrum indicates an interaction with *C. esculenta* AgNP, altering the polymer matrix. The spectral alterations provide compelling evidence that silver nanoparticles were effectively integrated into the chitosan-PEG patch, establishing stable interactions with the polymer’s functional groups (Alven and Aderibigbe, 2024).

#### 3.10.2 Physical appearance

**FIGURE 6:**
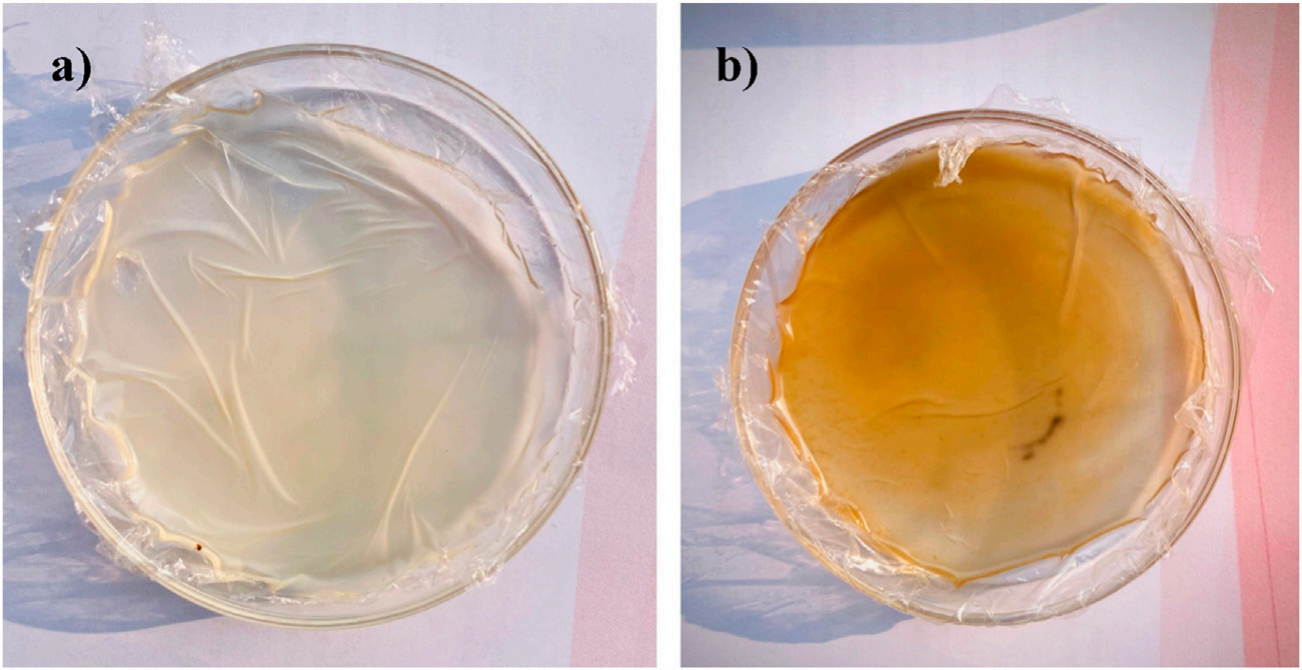
Physical appearance of **(a)** formulated chitosan patch and **(b)** *C. esculenta* AgNP-loaded chitosan patch.

Different concentrations of ingredients were employed to formulate the patches (F1-F6), as mentioned in Table 3. The physical appearance of the formulated chitosan patch and the *C. esculenta* AgNP-loaded chitosan patch was assessed by visual inspection of the prepared patches (Figures 6a,b). All formulated patches had a non-sticky, homogeneous, opaque, flexible, and smooth nature. In addition, the formulated patches exhibited even distribution of adhesive layer to diminish the lump formation. Furthermore, formulated patches exhibited the acceptable pH range (5.2–5.9). Therefore, the prepared transdermal patches are user friendly, as they will cause no irritation to the skin.

**TABLE 3:** Evaluation of various parameters of transdermal patches (mean ± SD).

Formulation	Thickness	Weight variation	Folding endurance	Tensile strength (Kg/cm^2^)	Elongation (%)	Moisture uptake (%)
F1	0.38 ± 0.12	0.073 ± 0.003	88 ± 2	0.61 ± 0.11	11.31 ± 0.15	0.31 ± 0.15
F2	0.43 ± 0.21	0.075 ± 0.006	97 ± 2	0.58 ± 0.13	14.51 ± 0.20	0.35 ± 0.10
F3	0.47 ± 0.19	0.078 ± 0.002	94 ± 3	0.60 ± 0.09	16.01 ± 0.12	0.38 ± 0.07
F4	0.46 ± 0.22	0.076 ± 0.007	96 ± 2	0.64 ± 0.03	14.39 ± 0.21	0.33 ± 0.20
F5	0.45 ± 0.11	0.070 ± 0.004	95 ± 3	0.66 ± 0.12	15.11 ± 0.09	0.34 ± 0.25
F6	0.46 ± 0.13	0.077 ± 0.001	96 ± 2	0.62 ± 0.08	12.91 ± 0.21	0.35 ± 0.11

Note: One-way ANOVA and *post hoc* Tukey’s test were employed to analyze the differences among formulations.

#### 3.10.3 Thickness uniformity

The thickness uniformity of the patches was observed by using the digital calipers, and the average thickness was investigated (Table 3). Chitosan concentration has an impact on the overall thickness of the patch, as it may result in a denser and more robust formulation of the matrix system of the polymer. The results suggest the optimum thickness uniformity in all prepared transdermal patches with the thickness order: F-1 < F-2 < F-3. However, no significant difference in thickness was observed in the formulations F4, F5, and F6.

#### 3.10.4 Moisture content

The moisture absorption profile of the prepared patches was investigated (Table 3) and showed the moisture absorption of the formulated patches in the order of F-3 > F-2 > F-1; however, formulations F-4 to F-6 have comparable moisture content due to similar CS concentrations. All formulations showed the moisture absorption in the acceptable range and retained an appropriate shape and texture.

#### 3.10.5 Uniformity of weight

The weight uniformity of the drug-loaded patches (1 × 1 cm^2^) was evaluated according to the lower SD values (Table 3). Patch weights were found to be uniform, as the thickness uniformity of the patches ensured the weight uniformity. As the concentration of the CS changes, achieving a uniform layer is difficult, and hence, the uniformity of weight may change accordingly.

#### 3.10.6 Folding endurance

All prepared transdermal patches exhibit favorable film properties and folding endurance, as shown in Table 3. The folding endurance of the prepared transdermal patches was found to be in the order of F-1 < F-2 > F-3, with the F2 formulation showing more folding endurance (97 ± 2). In addition, F4 and F6 exhibited the same folding endurance with value 96 ± 2, while F5 showed lesser folding endurance with value 95 ± 3. These results indicate that the folding endurance of the prepared patches increased with the corresponding increase in the CS concentration initially due to an increase in the elasticity and flexibility of the patch. However, as the concentration of CS increases above a certain value, it may decrease folding endurance due to the formation of a very rigid structure that reduces overall flexibility.

#### 3.10.7 Tensile strength

The tensile strength of the formulated patches was determined (Table 3) by employing the fabricated apparatus according to previous studies. Tensile strength was measured in triplicate, and the average was calculated. The tensile strength of the prepared patches increased with increased CS proportion, indicating that tensile strength can be increased with a high concentration of the polymer in the formulation.

#### 3.10.8 SEM analysis of transdermal patches

The surface morphology of the CS transdermal patches and CS patches (Figure 4b) loaded with *C. esculenta* AgNPs was analyzed using SEM to assess the impact of nanoparticle incorporation. Figure 4c displays the SEM image of the CS-based patch prior to *C. esculenta* AgNP incorporation, showing a smooth and uniform surface. The lack of particulate matter indicates a uniform polymeric structure, typical of a well-constructed CS-PEG film.

Figure 4c depicts the chitosan patch post-loading with *C. esculenta* AgNPs, revealing notable surface alterations. The formerly smooth surface now displays dispersed nanoparticle clusters, validating the successful integration of *C. esculenta* AgNPs into the polymeric matrix. The irregular and diverse morphology indicates significant interactions between *C. esculenta* AgNPs and CS, potentially affecting release dynamics and mechanical characteristics. These observations validate that nanoparticle incorporation modifies the patch morphology, shifting from a smooth surface to a rougher texture due to AgNP distribution, thereby validating the effective fabrication of a nanoparticle-loaded chitosan patch for prospective biomedical applications.

#### 3.10.9 Skin irritation and sensitization tests

A skin irritation test was done with the F4 formulation. A transdermal patch was employed on the skin of the experimental subjects, and subjects were examined after 24 h and 72 h. There was no sign of erythema and edema over the skin of experimental subjects. The primary irritancy index (PII) for the control patches and medicated patches was <2, while the PII for the formalin-treated group was 5.67 ± 0.52, indicating high irritation of the skin. Formulations with PII<2 are considered a non-irritant according to the Draize patch test. Therefore, patches formulated in this study were safe and non-irritant when applied to the skin for the specific time of application (Supplementary Figure S1).

These results demonstrate that the formulated transdermal patches (F4) are non-irritant and present good skin compatibility. The absence of any adverse reaction and irritation suggests that these patches are safe and reliable for prolonged application over the skin and are a suitable option for their intended transdermal drug delivery applications.

### 3.11 *Ex vivo* permeation

**FIGURE 7:**
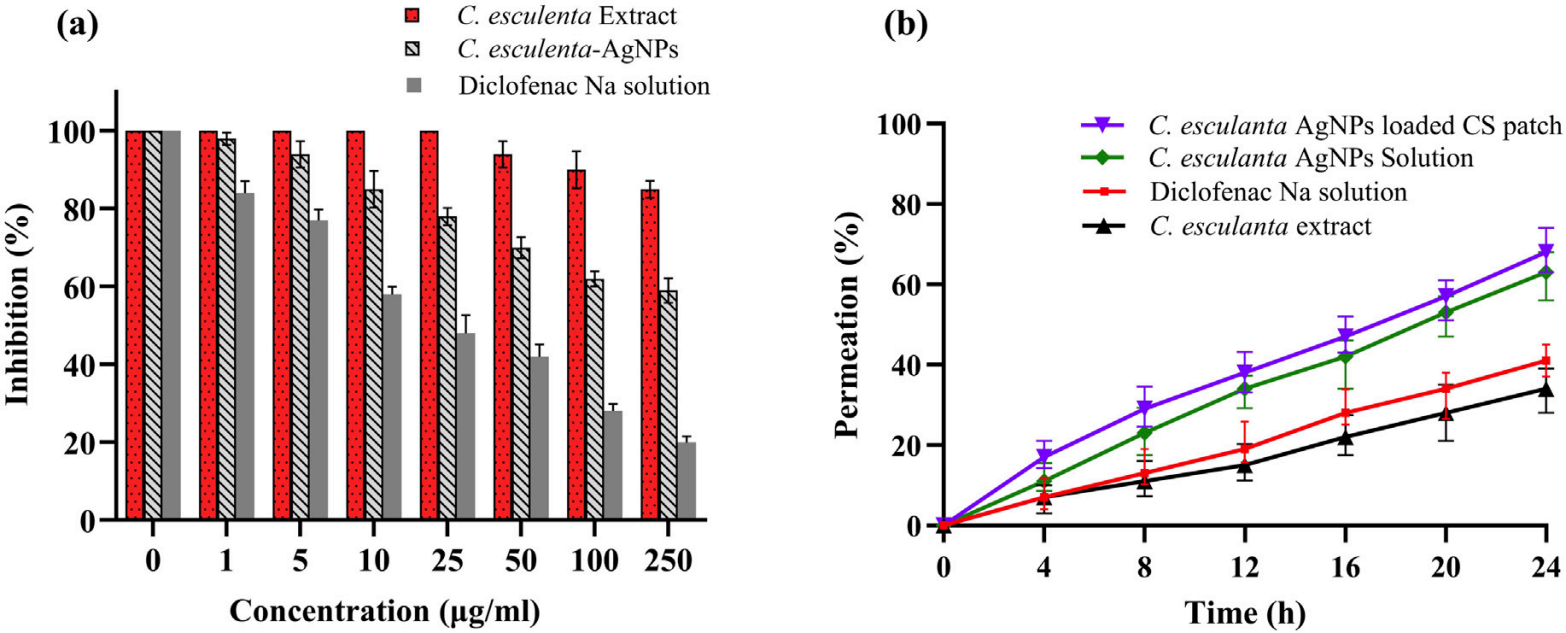
Protein denaturation effects of **(a)** control (diclofenac Na), *C. esculenta* extract, and *C. esculenta* AgNPs and **(b)** *ex vivo* permeation of *C. esculenta* extract, control (diclofenac Na), *C. esculenta* AgNP solution, and *C. esculenta* AgNP-loaded CS patches.

Using Franz diffusion cells and rat skin, an *ex vivo* permeation study was conducted on *C. esculenta* extract, *C. esculenta* AgNP, *C. esculenta* AgNP-loaded CS patches, and a control drug (diclofenac Na). Compared to the *C. esculenta* AgNP, the permeation of the CS patches loaded with *C. esculenta* AgNP was found to be significantly greater (ANOVA; p < 0.05). As a permeation enhancer, chitosan increases drug penetration by reversibly changing skin proteins and lipids (Nawaz and Wong, 2017). The results of the *ex vivo* permeation study are summarized in Figure 7b.

### 3.12 Egg albumin denaturation study

Protein denaturation is a recognized contributor to inflammation, and credible literature verifies the association between inflammatory and arthritic conditions and the denaturation of tissue proteins (OPIE, 1962; Williams et al., 2008). The results presented in Figure 7A indicate that *C. esculenta* crude extracts and their subsequent fractions demonstrate minimal inhibition of egg albumin denaturation. In contrast, *C. esculenta* AgNP exhibited the highest potency (80.1%, p < 0.001), followed by diclofenac Na (59.3%, p < 0.01), and *C. esculenta* crude extracts (15.1%, p < 0.01) at a concentration of 250 μg/mL, in a dose-dependent manner relative to the control. The results align with those reported by Chandra et al. (2012), demonstrating a concentration-dependent inhibition of egg albumin denaturation by experimental coffee extracts and standard diclofenac sodium. Other researchers demonstrated that the methanol extract of *Enicostemma axillare*, at concentrations ranging from 100 to 500 μg/mL, significantly safeguards against heat-induced protein denaturation (Leelaprakash and Mohan Dass, 2011). Our results indicate that *C. esculenta* AgNP (p < 0.001) demonstrates significant inhibition of serum albumin denaturation, followed by diclofenac Na (p < 0.001), and the crude extract of *C. esculenta* AgNP (p < 0.01), with percentage inhibitions of 80.1%, 59.3%, and 15.1%, respectively, at a concentration of 250 μg/mL compared to the control. Diclofenac sodium (p < 0.0001) demonstrated significant inhibition of egg albumin denaturation at a concentration of 250 μg/mL.

### 3.13 *In vivo* anti-inflammatory studies

#### 3.13.1 Determination of hematological parameters

The hematological parameters were assessed to evaluate the anti-inflammatory potential of the *C. esculenta* AgNP-loaded CS patches. The notable decrease in WBC count from 11.94 ± 0.92 × 10^9^/L prior to treatment to 6.94 ± 0.19 × 10^9^/L post-treatment signifies a reduction in systemic inflammation, given that elevated WBC levels are generally linked to an inflammatory response. Furthermore, neutrophil levels, essential in acute inflammation, exhibited a significant reduction from 28.11% ± 9.04% to 21.02% ± 7.11% following treatment, thereby corroborating the resolution of inflammation in the treatment group. The RBC count, initially lower before treatment (5.03 ± 0.31 × 10^6^/mm^3^), rose to 6.15 ± 0.22 × 10^6^/mm^3^ post-treatment, indicating enhanced hematological stability, presumably attributable to diminished oxidative stress and inflammation-related hemolysis. The platelet count, significantly elevated prior to treatment (1,610.98 ± 6.21 × 10^9^/L), decreased to 1,010.98 ± 4.78 × 10^9^/L following treatment, signifying a reduction in inflammatory and pro-thrombotic activity, given the pivotal role of platelets in immune responses and inflammation. Furthermore, the slight fluctuations noted in MCV, MCH, and mean corpuscular hemoglobin concentration (MCHC) indicate that the treatment did not negatively impact erythrocyte morphology or hemoglobin levels. The hematological parameters are presented in Table 4.

**TABLE 4:** Results of biochemical analysis of rat blood (Mean ± SD; n = 6).

Hematology/blood C/E	Group I (control)	Group III (before treatment)	Group III (after treatment)
Hb (10–15 g/dl)	13.20 ± 0.31	10.09 ± 0.17	12.09 ± 0.39
pH	7.29 ± 0.14	7.17 ± 0.31	7.28 ± 0.11
WBCs (×10^9^/L)	6.83 ± 0.18	11.94 ± 0.92	6.94 ± 0.19
RBCs (×10^6^/mm^3^)	6.04 ± 0.09	5.03 ± 0.31	6.15 ± 0.22
Platelets (×10^9^/L)	942.59 ± 3.75	1,610.98 ± 6.21	1,010.98 ± 4.78
Neutrophils (%)	20.03 ± 0.91	28.11 ± 9.04	21.02 ± 7.11
Lymphocytes (%)	72.57 ± 2.44	88.58 ± 1.34	74.08 ± 2.84
MCV (%)	54.39 ± 2.13	59.81 ± 2.24	55.30 ± 1.01
MCH (pg/cell)	16.88 ± 0.23	13.04 ± 0.82	15.10 ± 0.12
MCHC (g/dL)	29.82 ± 0.71	22.91 ± 1.07	28.07 ± 1.91

Note: The paired sample t-test was employed to analyze the pre-treatment and post-treatment values, while one-way ANOVA was employed to explore differences between the treated and control groups.

#### 3.13.2 Carrageenan-induced paw edema measurements

The anti-inflammatory effects of the prepared nanoparticle-loaded patches are illustrated in Supplementary Figure S2. Female Sprague–Dawley rats were employed in this study due to the prevalence of inflammation being 3–5 times higher in females than in males. *C. esculenta* is extensively documented as a potential anti-inflammatory agent owing to the presence of phenolic and flavonoid compounds (Sudhakar et al., 2020). The anti-inflammatory efficacy of *C. esculenta* phytochemicals was augmented by their transformation into nanoparticles to improve skin permeation. The results indicated that the highest percentage of inhibition in paw volume was recorded with patches containing nanoparticles (ANOVA; p < 0.05). Patches significantly suppress inflammation and edema relative to the control group. Diclofenac Na-loaded patches also diminish edema volume relative to the control, albeit to a lesser extent than the *C. esculenta* AgNP-loaded chitosan patches. This resulted from increased penetration of phytochemicals into skin layers due to enhanced skin permeation. The elevated concentration of nanoparticles in the deeper dermal layers aids in mitigating skin infections and inflammation.

The nanoparticle-infused patches administer the requisite quantity of anti-inflammatory agents at the target location with greater efficiency. Consequently, the topical administration of natural compounds via nanoparticles embedded in patches enhances the management of inflammation. The findings of anti-inflammatory studies are encapsulated in Table 5. The findings indicate that the treatment effectively reduced inflammatory responses and aided in restoring hematological homeostasis in the experimental model.

**TABLE 5:** Comparative study of different formulations on carrageenan-induced paw edema in rats (n = 5).

Treatment	Dose	Paw volume	% inhibition	T-value	P-value
Control	Nil	1.22 ± 0.54	Nil	Nil	Nil
*C. esculenta* AgNP-loaded CS patch	5% w/v	0.42 ± 0.05	62.99	−2.53	0.036**
Diclofenac Na-loaded patch	50 mg	0.98 ± 0.27	19.67	0.39	0.703

**indicates significant differences between controlled group and C. esculenta AgNP CS patch treated groups.

The anti-inflammatory effect found in the study is predominantly due to the silver nanoparticles (AgNPs). Several studies described that AgNPs exhibit anti-inflammatory characteristics as they inhibit the pro-inflammatory cytokines (e.g., IL-6 and TNF-α), modulate the NF-κ signaling pathway, and suppress the reactive oxygen species (ROS) (Gopinath et al., 2010; Liu et al., 2014). AgNPs may release some silver ions (Ag+); they typically act synergistically and may contribute to the anti-inflammatory effect. In our study, *C. esculenta*-mediated AgNPs incorporated into the CS patches exerted an active anti-inflammatory effect.

In the present study, the CS-based matrix played a pivotal role in the transdermal mechanism as CS is a bioadhesive, biocompatible, and permeation-enhancing polymer, having the ability to open the tight junctions present in the skin, thus facilitating the paracellular transport. CS also promotes the intimate contact between skin and the applied patch, thus enhancing the absorption through the skin.

AgNPs may penetrate the upper layer of the skin more efficiently, owing to their nano-size, further facilitating the skin permeability. These factors demonstrate the efficient and sustained transdermal delivery of the active constituents via a combination of NP-assisted penetration and polymer-aided permeation enhancement.

## 4 Conclusion

The present research successfully demonstrated the green production of silver nanoparticles (AgNPs) using *C. esculenta* extract, which were then integrated into CS-based transdermal patches for anti-inflammatory purposes. The synthesized AgNPs were characterized by studies that confirmed their stability, spherical form, and good physicochemical properties. Transdermal patches showed great potential as an effective medication delivery system due to their high levels of biocompatibility, mechanical integrity, and sustained drug release patterns. The *in vivo* and *in vitro* studies demonstrated that the anti-inflammatory effects of the patches loaded with AgNPs were significantly stronger than those of the usual diclofenac sodium formulations. Superior efficacy in moderating inflammatory responses is shown by the considerable suppression of protein denaturation and reduction in carrageenan-induced paw edema, which are both caused by AgNPs. In addition, the chitosan matrix allowed for better drug dispersion, which was confirmed by the *ex vivo* permeation experiments, which led to improved transdermal penetration. Additional evidence of the formulation’s safety is the lack of skin irritation and sensitization.

These results highlight the possibility of AgNPs derived from *C. esculenta* as a greener, more sustainable substitute for current anti-inflammatory treatments. To determine the therapeutic feasibility of these new transdermal patches, additional research is needed to examine their long-term toxicity, clinical translation, and the anti-inflammatory pathways from a molecular perspective.

## 5 Limitations

The present study utilized zeta potential studies for stability, SEM for surface morphology, and dynamic light scattering (DLS) for size and PDI measurement. However, nanoscale imaging studies, such as atomic force microscopy (AFM) and transmission electron microscopy (TEM), could provide better insights for the evaluation of surface topography and NP morphology.

## 6 Future perspectives

Although *C. esculenta*-mediated AgNPs exhibit significant anti-inflammatory potential, the immunomodulatory role of *C. esculenta*-mediated AgNPs has yet to be mechanistically explored. The LPS-induced macrophage model, which uses RAW 264.7 cells to assess TNF-α, IL-6, and IL-1β concentration, will assist in elucidating the cellular pathways involved in future studies. These assays would enlighten the understanding of the biological activity of the AgNPs and complement the current findings. While the colloidal stability of AgNPs was not assessed in PBS or FBS due to solid-state patch formulation, *C. esculenta*-AgNP stability under physiological conditions may be investigated in future research.

## Data Availability

The original contributions presented in the study are included in the article/Supplementary Material, further inquiries can be directed to the corresponding author.
