# Supplementary material for: Green synthesis of Colocasia esculenta-based silver nanoparticles: characterization and transdermal delivery for anti-inflammatory applications
Source: Front Pharmacol. 2025 Aug 14;16:1611507. doi: 10.3389/fphar.2025.1611507 (PMC12391045; doi:10.3389/fphar.2025.1611507)
Supplement: Supplementary file 1 [file DataSheet1.docx]

**Supplementary Material**

**Green Synthesis of *Colocasia esculenta*-based Silver Nanoparticles: Characterization and Transdermal Delivery for Anti-Inflammatory Applications**

Xiaobo Wang^1^, Wanjuan Wang^2^, Areeba Ejaz^3^, Maira Mehmood^3^, Nadeem Ahmad^3^, Imran Nazir^3^, Yasser Shahzad^3^, Tianshuo Bai^4*^

^1^Department of Dermatology, Hejin Wang Xiaobo Dermatology Clinic, Hejin, 043300, China

^2^Department of Dermatology, The Second Affiliated Hospital of Xi'an Jiaotong University (Xibei Hospital), Xi'an, 71000, China

^3^Department of Pharmacy, COMSATS University Islamabad, Lahore Campus, Lahore,54000, Pakistan

^4^Department of Emergency, XD Group Hospital, Xi 'an, 710086, China


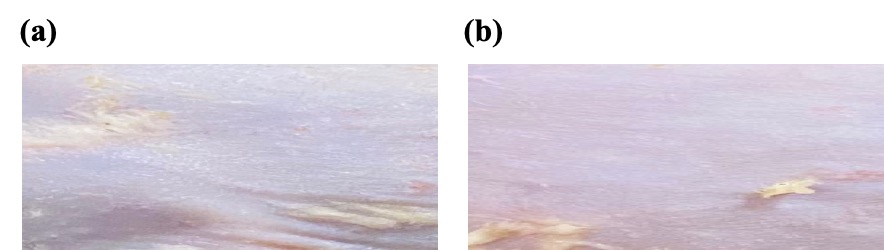


**Fig S-1. (a) Skin of the control animal (b) C. esculenta Ag-NPs loaded Chitosan patch applied skin**


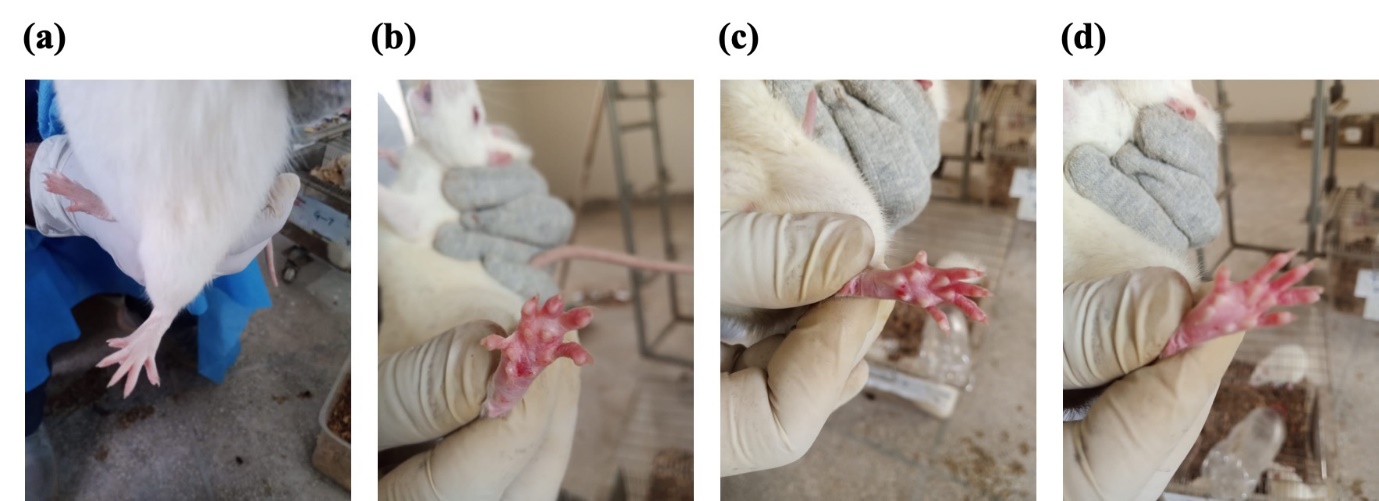


**Fig S-2. (a) Paw of the normal Control (b) Paw of the Control with edema (c) Diclofenac Na containing Chitosan patch treated Paw (d) C. esculenta Ag-NPs loaded Chitosan patch treated Paw**
